# Human bone graft cytocompatibility with mesenchymal stromal cells is comparable after thermal sterilization and washing followed by γ-irradiation: an *in vitro* study

**DOI:** 10.1093/rb/rby002

**Published:** 2018-01-31

**Authors:** Dmitry Labutin, Konstantin Vorobyov, Svetlana Bozhkova, Ekaterina Polyakova, Tatyana Vodopyanova

**Affiliations:** Division of Wound Infection Treatment and Prevention, Vreden Russian Research Institute of Traumatology and Orthopedics, St. Petersburg, Russian Federation

**Keywords:** bone grafts, bone sterilization, mesenchymal stromal cells, biocompatibility testing

## Abstract

Human bone allografts present a better alternative to autografts in terms of minimization of the harvesting procedure complications. Prior to the use in clinical applications, they require sterilization which aims to reduce bioburden. This often comes at the expense of their biological properties as carriers of cells. In this study, we evaluated the cytocompatibility of human bone allografts processed and sterilized by three different methods with mesenchymal stromal cells. Bone morphology, biological and biochemical properties of the extracted bone-conditioned medium and viability of cells were assessed. We found that chemical sterilization had a strong negative effect on cell viability, whereas thermal sterilization and washing with subsequent γ-irradiation both resulted in a bone graft compatible with the progenitor cells. Moreover, washing of the bone prior to sterilization allowed solid removal of cell debris and other bone marrow components. Taken together, our findings demonstrate the importance of a proper choice of the bone graft processing method for the production of the biomaterial suitable for tissue engineering.

## Introduction

Grafting of the human bone is extensively employed in orthopedics and maxillofacial surgery to promote the repair of bone tissue. This includes the treatment of non-union fractures, augmentation of large bone defects and restoration of the bone stock in revision surgery for periprosthetic osteolysis [1–3]. Depending on the source, natural human bone grafts can be either of two types: autogenous or allogenous. Bone autografts are usually harvested from the iliac crest, fibula or ribs of the same patient receiving the graft. Promotion of osteoinduction and osteogenesis by bone autografts is attributed to growth factors and progenitor cells carried by them [4]. Although autografts remain the gold standard material for bone augmentation, their broad clinical usage is hampered by frequent complications after the harvesting which often results in the insufficient amount of the procured bone to meet clinical demand. The most common complications include infection, blood loss, neurovascular damage and chronic surgical site pain [5]. In contrast to autografts, allografts are a safer alternative as they can be sourced in large quantities from cadaveric or live donor bones. Their main disadvantage is a more complex processing procedure which aims to prepare the bone for a long-term storage, eliminate infectious pathogens and prevent graft rejection due to the potential immunogenicity of donor cells. Consequently, this process leads to the loss of osteoinductive and osteogenic properties of the allograft as the obtained material becomes a scaffold devoid of proteins and cells.

Mesenchymal stromal cells (MSCs) are clonogenic adult progenitor cells which can be isolated from bone marrow, adipose tissue, dental pulp, synovium, periosteum and other tissues [6]. They are characterized by fibroblast-like growth, adherence to plastic and expression of a distinct set of CD markers: positive for CD44, CD73, CD90, CD105 and negative for CD11b, CD14, CD45. Under specific *in vitro* conditions, these cells can also differentiate toward at least three lineages: osteogenic, chondrogenic and adipogenic [7]. MSCs were shown to enhance repair of the bone defect upon local delivery but their exact role in the bone healing process is still poorly understood [7, 8]. Contrary to the expected direct involvement of the osteogenically differentiated MSCs at the site of damage, their contribution appears to be primarily via secretome containing growth factors which recruit osteoprogenitor cells from stem cell niches [9–11]. Therefore, to present a viable alternative to autografts, a combination of bone allografts with MSCs could be beneficial for the restoration of their osteoinductive and osteogenic properties. This could only be possible if the processing techniques are optimized to preserve the biocompatibility of bone allografts.

Bone allograft processing generally includes the removal of residual tissues surrounding the recovered bone and irrigation followed by disinfection or terminal sterilization. Although being effective in bioburden reduction, sterilization methods are reported to degrade not only integrity but also biocompatibility of allografts. In particular, bacterial debris and cellular components remaining inside the graft after sterilization may have a detrimental effect on osseointegration process [12, 13]. Lomas *et al.* [14] previously described a wash protocol for the recovered donor bone which since then has been optimized for better removal of protein, fat, blood and bone marrow [15, 16]. Current scientific data on the biological performance of washed and γ-irradiated bone allografts as scaffolds for MSCs in comparison to other processing methods is limited.

Bone-replacement materials based on calcium sulphate, calcium phosphate, hydroxyapatite or silicate could serve as another alternative to bone autografts [17, 18]. Clinically approved synthetic substitutes usually come in ready-made kits which apart from component mixing do not require any special preparation or labor-intensive processing [19]. Due to the presence of interconnected pores, these materials can be used as carriers for progenitor cells. However, chemical composition might affect their compatibility with cells. Several calcium phosphate and hydroxyapatite products demonstrated inferior adherence and metabolic activity of human MSCs as compared with bone allografts [20]. Moreover, brittleness of calcium phosphate cements and rapid resorption rate of calcium sulphate limit their use in load-bearing applications [21–23].

The main objective of our study was a pre-clinical evaluation of cytocompatibility of human bone allografts processed and sterilized by three different methods (chemical, thermal and washing with subsequent γ-irradiation) with MSCs.

## Materials and methods

### Processing and sterilization of the procured bone

The study was approved by the Institutional Ethics Board and informed consents were obtained from the donors. Femoral heads were harvested intraoperatively from patients (age range 53–68 years) undergoing total hip arthroplasty. The procured bone material was stored at −80°C in sterile polyethylene containers. Later, batches of collected femoral heads were processed in a biological safety cabinet MSC-9 (Thermo Fisher Scientific, USA). After thawing for 8 h at room temperature and removal of extraneous tissues, the heads were cut with a medical hand saw into blocks (cubes) with the average side length of 0.5 cm. The blocks were incubated overnight at 4°C in 400 ml saline containing 1.0 g of ciprofloxacin. After that, the blocks were processed and sterilized with three different methods.

Chemical sterilization protocol included washing of the blocks in 3.0% sodium hydrocarbonate solution with a brush and soaking in saline for 30 min. After drying at room temperature, bone blocks were packed in polyethylene bags filled with a disinfectant solution and stored at −20°C. The solution contained a mixture of citric acid, glucose, sodium bromide, 95% ethanol, nitrofural, dimethyl sulfoxide (DMSO), amikacin in distilled water (Patent RU2235462).

Thermal sterilization involved heating of the bone blocks placed into a sealed container with saline in Lobator SD-2 sterilizer (TELOS, Germany). The duration of the protocol was 94 min, while sterilization temperature (82.5°C) was sustained for at least 15 min. After cooling, saline was discarded via a safety valve and the container was stored at −20°C. This sterilization procedure is described in more detail elsewhere [24].

Processing of the bone blocks by washing with γ-irradiation was based on a previously published protocol and included our modifications [25]. Briefly, bone blocks were agitated in distilled water for 60 min at 59°C and cleaned in hydrodynamic water flow for 15 min. After that, three consecutive wash cycles were performed.

The first wash cycle consisted of 20 min in 10% sodium hydrocarbonate, 15 min in hydrodynamic flow, 20 min of sonication (45 Hz) in 10% sodium hydrocarbonate at 59°C, 15 min in hydrodynamic flow, 15 min in 10% sodium hydrocarbonate with agitation at 220 rpm, 15 min centrifugation at 1850 g.

The second wash cycle consisted of the same steps as previous only with distilled water instead of sodium hydrocarbonate. A total of five repeats were performed.

The third cycle included washing in 3% hydrogen peroxide at 59°C in a shaking water bath (22), sonicator (45 Hz) and orbital shaker (220 rpm) for 20 min each. After that, the same wash sequence was completed with 70% ethanol.

Finally, bone blocks were agitated three times for 15 min in sterile distilled water at 59°C. The blocks were dried for 2 h at 45°C and quick-frozen at −80°C. Later, the blocks were lyophilized on HETO PowerDry PL3000 (Thermo Fisher Scientific, USA) for 40 h, packed in double polyethylene bags (Clinipack, Russia) with the average length of 5 cm and γ-irradiated on a conveyor belt with a dose of 25 kGy at a speed of 150 cm/min. The resulting average exposition time was 2 s. Packed sterile blocks were stored at −20°C.

### Cell culture media

Standard growth medium: low-glucose DMEM GlutaMAX, 15% fetal calf serum (FCS), penicillin 100 U/ml—streptomycin 100 µg/ml (Life Technologies, UK).

Osteogenic medium: low-glucose DMEM GlutaMAX, 15% FCS, penicillin 100 U/ml—streptomycin 100 µg/ml, 10 mM β-glycerophosphate disodium salt hydrate, 10 nM dexamethasone, 50 µg/ml L-ascorbic acid (Sigma-Aldrich, USA).

Chondrogenic medium: low-glucose DMEM GlutaMAX, 1% FCS, penicillin 100 U/ml—streptomycin 100 µg/ml, 1X ITX-G (Life Technologies, UK), 50 µg/ml L-proline, 100 nM dexamethasone, 50 µg/ml L-ascorbic acid (Sigma-Aldrich, USA), 10 ng/ml recombinant human TGFβ3 (Miltenyi Biotec, Germany).

Cells were grown in the atmosphere of 5% CO_2_ unless otherwise specified.

### Bone-conditioned medium

Bone blocks were placed into 50 ml centrifuge tubes containing the standard growth medium without FCS. The amount of medium was 2 ml per block with a total of 10 blocks per tube. Samples were incubated at 37°C with constant agitation at 200 rpm. In 24 h, the medium was collected and centrifuged at 2000 rpm for 10 min. The obtained supernatant was aliquoted into sterile 15 ml tubes and stored at −20°C.

### Protein concentration and pH of BCM

Protein concentration in bone-conditioned medium (BCM) was determined according to manufacturer’s instructions on Qubit 3.0 Fluorometer using Qubit Protein Assay Kit (Thermo Fisher Scientific, USA). The amount of protein in the control medium was subtracted from sample values to obtain final concentrations. Samples of the serum-free medium served as a negative control. Bone blocks were incubated in the stock medium and pH was measured with pH METER 410 (Akvilon, Russia) at 0 and 24 h.

### Scanning electron microscopy

Processed bone blocks were fixed overnight in 3.7% formaldehyde solution at 4°C. Next day, blocks were briefly washed with PBS and dehydrated in a series of increasing concentrations of ethanol: 35, 50, 70, 100% (each step 15 min). Samples were dried, sputter coated with gold/palladium particles and imaged on JSM 6390LA microscope (JEOL, Japan).

### Isolation and expansion of MSCs

Animal experiments were performed in compliance with the local and institutional regulations. MSCs were isolated from bone marrow of 4 weeks old female Wistar rats. Femurs were harvested from the euthanized animals and kept on ice. After removal of the proximal and distal ends of the femurs, bone marrow was flushed out with PBS into separate 15 ml tubes which were briefly vortexed and centrifuged for 5 min at 1500 rpm. The supernatant was discarded and cells were resuspended in 10 ml of the standard growth medium. The concentration of cells was determined on Countess II FL Automated Cell Counter (Thermo Fisher Scientific, USA). A total of 6 × 10^6^ cells were seeded into each well of a 6-well tissue culture plate and incubated overnight at 37°C. The next day, the growth medium was replaced with the fresh one and cells were let to reach 80% confluence. After that, cells from each well were transferred into separate T75 tissue culture flasks. Every 3–5 days, cells were washed with PBS, detached with TrypLE Express (Life Technologies, UK), split 1:3 and supplemented with fresh growth medium until P3. Isolated cells were frozen in FCS with 10% DMSO (Sigma-Aldrich, USA) and stored at −80°C. The phenotype of MSCs was confirmed at P4 on CytoFLEX Flow Cytometer (Beckman Coulter, USA) using a panel of antibodies: mouse anti-rat CD45 PE, mouse anti-rat CD90 FITC (BD Pharmingen, USA). MSCs were identified as a population of at least 95% of CD90+CD45-cells.

### Differentiation of MSCs

MSCs were cultured in the osteogenic medium at 37°C in a 24-well tissue culture plate at a density of 100 × 10^3^ per well for 14 days. The medium was replaced with the fresh one every 3 days. Osteogenesis was evaluated with Alizarin Red S (ARS) staining of the cells for the presence of calcium deposits. Briefly, cells were washed once with PBS and fixed in 3.7% formaldehyde for 10 min. After being washed twice with ddH_2_O, cells were stained with ARS solution (pH 4.3) for 10 min. Following that, cells were washed once with ddH_2_O and twice with PBS. Images of wells were acquired at 4× magnification on EVOS FL microscope.

For chondrogenic differentiation, MSCs were seeded at a concentration of 100 × 10^3^ in a 96-well tissue culture plate and grown in the chondrogenic medium at 37°C for 21 days. The medium was replaced with the fresh one every 3 days. Chondrogenesis was evaluated with Real-time PCR (RT-PCR). Cells were lysed in Trizol (Thermo Fisher Scientific, USA) for 10 min at room temperature. RNA isolation with subsequent cDNA reverse transcription was performed according to manufacturer’s instructions with RNA Extraction Kit (Biosilica, Russia) and OT-1 Kit (Syntol, Russia), respectively.

RT-PCR reaction was performed in 15 µl volume with 1 ng/µl of cDNA on CFX96 Touch TM Real-Time PCR cycler (Bio-Rad, USA) using SYBR Green PCR Mix (Syntol, Russia). The cycling conditions were 95°C for 4 min 45 s, then 40 cycles: 95°C for 30 s, 57°C for 45 s, 72°C for 15 s and final 72°C for 10 min. Primers were designed with NCBI Primer-BLAST software. Their sequences were as follows (5′–3′): *ACAN*-F-GAGAACCGTCTACCTCTACCXXX, *ACAN*-R-TACCTCGGAAGCAGAAGG, *18S*-F-CATTCGAACGTCTGCCCTAT, *18S*-R-GTTTCTCAGGCTCCCTCTCC. The fold change of *ACAN* expression to untreated control was determined relative to *18S rRNA* with a ΔΔCT method.

### MTT viability assay

MSCs were seeded at a concentration of 3 × 10^3^ cells per well in a flat bottom 96-well plate and grown overnight at 37°C in the standard medium. In 24 h, the medium in each well was replaced with 200 µl of BCM. Untreated control cells were kept in the standard medium without FCS. After 72 h of incubation, MTT stock solution (MP Biomedicals, USA) was added into each well to a final concentration of 1 mg/ml. Following incubation at 37°C for 3 h, the medium with MTT was discarded and wells were filled with 100 µl of DMSO. Plates were incubated in a shaker at 37°C and 200 rpm for 10 min. The absorbance at 540–620 nm was measured on Thermo iEMS Reader (Thermo Fisher Scientific, USA) and corrected by subtraction of the background DMSO absorbance from the averaged technical replicates. Ratio of MSC viability was calculated as BCM-treated sample absorbance divided by the control sample absorbance.

### Live/dead cell viability assay

MSCs were seeded at a concentration of 3 × 10^3^ cells per well in a flat bottom 96-well culture plate and grown overnight at 37°C in the standard medium. In 24 h, the medium was replaced with 200 µl of BCM per well. Untreated control cells were kept in the standard medium without FCS. After 72 h of incubation, the medium was replaced with 100 µl of PBS solution containing 2 drops/ml of NucBlue Live and NucGreen Dead reagents from Ready Probes Cell Viability Kit (Life Technologies, UK). Cells were incubated at 37°C for 15 min and imaged on EVOS FL fluorescent microscope (Thermo Fisher Scientific, USA) at 4× magnification using DAPI/GFP filters. Percentage of dead cells (green) was calculated in relation to the total number of cell nuclei (blue) using cell counter plug-in in ImageJ Fiji1.51n software.

### Viability of MSCs in bone blocks

Bone blocks were thawed and placed into 24-well plates. Prior to seeding, blocks after chemical sterilization were briefly flushed with 5 ml PBS. A total of 250 × 10^3^ MSCs in 100 μl was seeded on the blocks using 200 µl pipette tips. Samples were incubated at 37°C for 30 min. Bone blocks without cells served as a negative control. After that, the wells were filled with 1.5 ml of the standard growth medium. Bone blocks with MSCs were incubated for 3 and 7 days. In case of 7-day incubation, blocks were transferred on the third day with sterile forceps into a new 24-well plate filled with fresh standard medium. At the end of the incubation period, MTT stock solution was added to the wells for a final concentration of 1 mg/ml. Plates were kept at 37°C for 3 h. Bone blocks were air dried on a paper filter for 15 min. Thereafter, they were placed into a 24-well plate with 1.5 ml of DMSO in each well. Following the incubation in a shaker at 37°C and 200 rpm for 10 min, 100 µl of the obtained solution was transferred to a 96-well plate. The absorbance was measured at 540–620 nm on Thermo iEMS Reader (Thermo Fisher Scientific, USA).

### Statistics

All experiments were performed in biological replicates (*n* ≥ 3). The data is shown as means with standard deviation. The difference between means of experimental groups was evaluated with GraphPad Prism 6.0 (USA): one-way ANOVA with Tukey *posthoc* test for the analysis of independent protein concentrations and pH values, repeated measures two-way ANOVA with Sidak *posthoc* test for the analysis of paired pH values, two-tailed unpaired Welch’s *t*-test for all the comparisons of cell viability to the control group. Differences between means were considered significant if *P* values < 0.05.

## Results

### MSC lineage commitment

**Figure 1:**
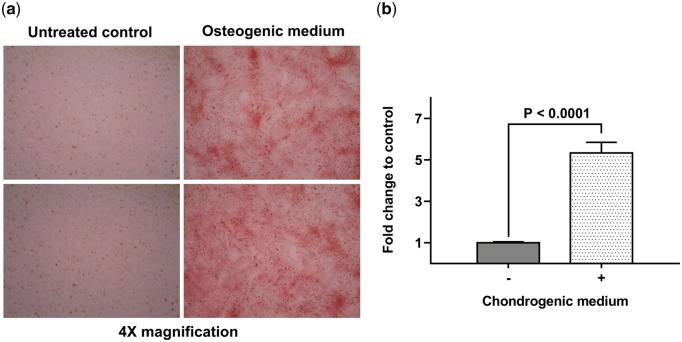
Differentiation of MSCs. (**a**) ARS staining of MSCs grown in the osteogenic medium for 14 days (right) and untreated cells (left). All pictures were taken at 4× magnification. (**b**) Results of RT-PCR: fold change of *ACAN* expression in MSCs grown in the chondrogenic medium for 21 days compared with untreated cells

After 14 days of MSC growth in osteogenic medium, there was a positive ARS staining indicating deposition of calcium (Fig. 1a). At the 21st day of chondrogenesis, there was a 5-fold increase of *ACAN* expression suggesting the production of the extracellular matrix (Fig. 1b).

### Morphology of sterilized bone blocks

**Figure 2:**
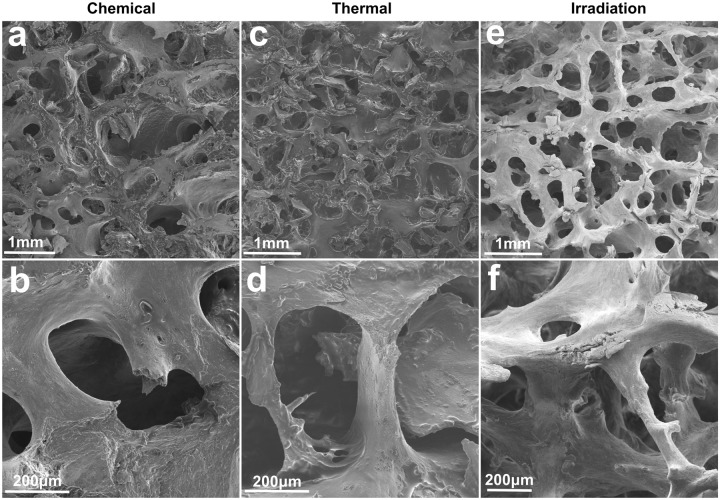
SEM of the bone blocks after processing and sterilization

Scanning electron microscopy (SEM) of the bone blocks showed a relatively dense network of rod-like and plate-like trabeculae in all types of samples. The surface of trabeculae in bone blocks after washing and γ-irradiation appeared to be cleaner as opposed to the ones sterilized by other methods (Fig. 2e and f). Samples after chemical (Fig. 2a and b) and thermal sterilization (Fig. 2c and d) both had the apparent presence of destroyed bone tissue components covering trabeculae and partially filling the intertrabecular space.

### Biochemical properties of BCM

The obtained results are depicted in Table 1. Protein level in BCM from bone allografts after thermal sterilization and γ-irradiation was ∼4 times lower than in the medium after chemical sterilization. The level of BCM pH in all samples showed the alkaline state. There was an alkaline shift of pH over 24 h incubation period. At 24 h, pH of BCM after chemical sterilization was lower as compared with thermal sterilization and γ-irradiation.

**Table 1:** Biochemical properties of BCM

Type of BCM	pH			Protein concentration (µg/ml)
	0 h	24 h	*P* value	
Chemical	8.0 ± 0.11	8.2 ± 0.1*	<0.01	164 ± 20*
Thermal	8.5 ± 0.04	8.6 ± 0.02	0.02	44 ± 23
γ-Irradiation	8.4 ± 0.03	8.6 ± 0.02	<0.01	40 ± 16
Control medium	8.5 ± 0.04	8.9 ± 0.07	<0.0001	N/A

Reported *P* values in the column correspond to the comparison of pH levels of each BCM type at 0 and 24 h;

* *P* < 0.0001 refers to the comparison of BCM pH levels and protein concentrations between samples: chemical vs. thermal and chemical vs. γ-irradiation.

### Effect of BCM on MSC viability

**Figure 3:**
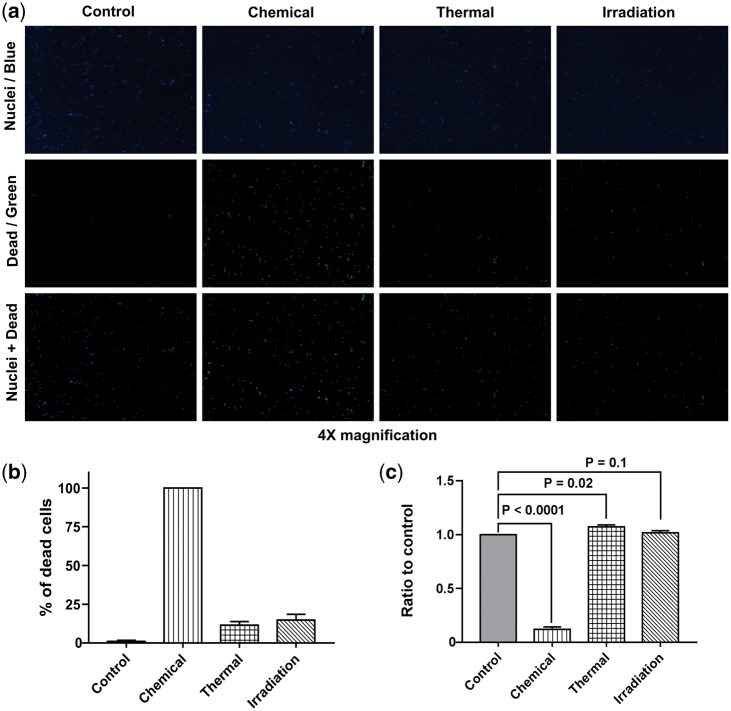
Results of MSC viability assays with BCM. (**a**) Representative images of MSC fluorescent microscopy after exposure to BCM for 72 h with (**b**) the calculated percentage of dead cells. All pictures were taken at 4× magnification. (**c**) MTT assay: ratio of MSC viability normalized to control after 72 h culture with different types of BCM

Representative images of live/dead fluorescent staining of MSCs after 72-h growth in tested BCM are shown in Fig. 3a. The calculated proportion of dead cells is depicted in Fig. 3b. The proportion of dead MSCs grown in control BCM was 1.1 ± 0.7% whereas in chemical BCM it was 100.0%. At the same time, fractions of dead MSCs cultured in BCM from bone blocks after thermal sterilization and γ-irradiation were 11.5 ± 2.3% and 14.8 ± 3.7%, respectively.

Results of MTT assay for MSCs grown in BCM for 72 h are shown in Fig. 3c. Mean ratio of MSCs viability cultured in BCM after chemical sterilization was 0.12 ± 0.02, whereas in case of BCM after thermal sterilization and γ-irradiation it was 1.07 ± 0.02 and 1.02 ± 0.02, respectively.

### Viability of MSCs after growth in bone blocks

**Figure 4:**
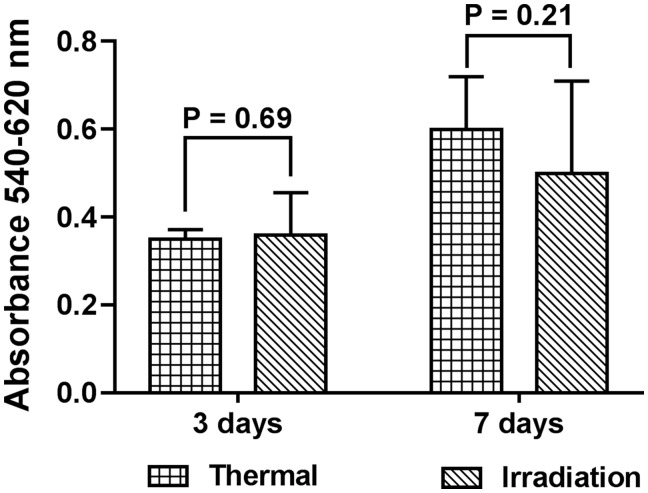
Results of MTT assay for MSCs grown in bone blocks for 3 and 7 days

Results of MTT test showed comparable absorbance of formazan after MSC growth in bone blocks. The absorbance values for thermal sterilization and γ-irradiation were 0.352 ± 0.022 and 0.363 ± 0.116 at 3 days, 0.601 ± 0.116 and 0.497 ± 0.207 at 7 days, respectively (Fig. 4).

## Discussion

Although allograft bone represents a promising alternative to the autograft, it lacks essential qualities of osteoinductivity and osteogenesis. Most of the bone processing and sterilization methods not only degrade osteoconductivity of bone allografts but also impair their biocompatibility. This hinders the possibility to enhance osseointegration of bone allografts via their enrichment with pro-osteogenic progenitor cells such as MSCs.

Our study focused on the changes of morphological, biochemical and biological properties of bone allografts prepared by three different processing techniques. The main purpose of all three methods is the reduction of bioburden. It is achieved by means of either a cocktail of antibiotics with chemical sterilants or exposure to the temperature of at least 82.5°C for 15 min or γ-irradiation with a 25 kGy dose which is harmful to pathogens. In addition, these techniques help to preserve the harvested bone for long-term storage. Extensive cyclic washing of the bone grafts in combination with γ-irradiation allows achieving bioburden reduction with the added benefit of protein, lipid and cellular debris removal [25].

Bone grafts carry numerous proteins which regulate the process of osseointegration via paracrine mechanisms [26]. Peng *et al.* [27] showed that BCM from porcine bone chips inhibits osteogenesis of murine stromal cell line ST2 via activation of ERK signaling pathway downstream of TGF-β receptor. Thus, the presence of endogenous proteins could complicate standardization of protocols for bone graft enhancement with growth factors and negatively affect the differentiation of progenitor cells. Taking this into account, we analysed the concentration of proteins in BCM which was expectedly lower after cyclic washing with γ-irradiation as opposed to the chemical processing method. Sterilization of the bone blocks by the heat also reduced the protein content of BCM to a similar extent as washing (Table 1). Additionally, SEM revealed the presence of tissue debris only in bone blocks processed with the chemical and thermal method (Fig. 2a–d), supporting the efficacy of bone marrow component removal by the washing procedure (Fig. 2e and f).

We further evaluated the effect of BCM on MSC metabolism. Given common understanding that results of MTT assay do not directly correspond to the number of viable cells, we also estimated the percentage of dead cells in a fluorescent live/dead staining. The extract from chemically sterilized bone markedly decreased metabolic activity of cells and rendered 100% of them dead as demonstrated by the staining. Although this corroborates previously reported reduction of MSC viability by BCM from fresh-frozen bone [28], we believe that traces of chemical sterilants such as sodium bromide, ethanol or DMSO rather than marrow components were responsible for cell death. Despite stimulation of cell viability by BCM after washing with γ-irradiation demonstrated in the same report, we did not observe any significant changes of MSC metabolic activity with this method as opposed to untreated cells. Conversely, incubation of MSCs in BCM from heat-sterilized bone resulted in a slight increase of cell metabolism whereas the percentage of dead cells was similar to that of the extract from washing with γ-irradiation. This suggests possible activation of MSC differentiation process. In fact, Caballé-Serrano *et al.* [29] showed that porcine BCM pre-heated for 10 min at 85°C enhances osteoclastogenesis in murine bone marrow cells. The authors hypothesized that either activation of growth factors or inactivation of inhibitors triggers the cells. Furthermore, analysis of MSC seeded on the bone blocks did not reveal any substantial difference in cell metabolic activity between thermal sterilization and washing with γ-irradiation. Previously, Endres *et al.* [30] reported significantly decreased percentage of viable mononuclear cells after 28-day growth in bone discs sterilized by 25 kGy γ-irradiation. Considering that the wash protocol employed in that study was less intensive than in our preparation of the bone grafts, γ-irradiation most likely resulted in lipid peroxidation with the formation of free radicals toxic to cells [31].

Excessive alkalinization of the local cellular environment has been reported to decrease the viability of MSCs (pH > 8.27) and inhibit their ability to differentiate toward the osteogenic lineage (pH > 7.9) [32]. We assessed pH of BCM from the bone blocks and found that it was in the alkaline state (Table 1). The pH levels of the extracted medium were within 8.2–8.6 range while chemical sterilization resulted in the lowest pH (8.2). Taking together our MSC viability data, the fact that we measured pH of the conditioned medium after 24 h incubation without cells and the alkaline state of the control medium, we are unable to confirm previously reported effect of alkaline pH on MSC metabolism. It should also be noted that suggested pH thresholds (7.9 and 8.27) were taken from the study focusing on the inorganic bone-replacement material which unlike natural bone usually releases chemical compounds for a longer period of time.

Although it is apparent from our data that cyclic washing with γ-irradiation should be a preferable way of bone scaffold production for tissue engineering applications, the choice of the bone graft processing and sterilization protocol should be based on the needs for a clinical situation. For instance, thermal sterilization would be beneficial in scenarios when a large amount of bone material is required in a short period of time. Cyclic washing of the bone with sterilization by γ-irradiation would be preferable in case of the bone critical size defect repair when the delivery of a scaffold with a standardized dose of MSCs or pro-osteogenic growth factors is advantageous. In addition, the ability of a medical facility to house and maintain specialized equipment (e.g. irradiation machines, low-temperature freezers) should be taken into account. Although being unsuitable for tissue engineering applications, the chemical sterilization method could be a cost-effective alternative to more complex procedures.

We acknowledge the following limitations to our study: (i) this was a pre-clincal *in vitro* evaluation of human bone grafts and rat MSCs, thus the use of cells from different species could have a negative effect on the reproducibility of the data in further studies with human cells; (ii) the obtained results showed *in vitro* activity of MSCs which would not necessarily be the same after their delivery on the bone grafts into a living organism; (iii) a slight degree of variability in the volume of the tested block is expected due to the use of a hand saw; (iv) since manufacturer’s instructions for thermal sterilization are only valid for whole femoral heads, we report exposure to at least 82.5°C as it was not possible for us to determine the actual temperature inside the bone blocks.

## Conclusions

We have shown that thermal sterilization and washing with subsequent γ-irradiation do not exhibit any significant effects on the cytocompatibility of human bone grafts with MSCs, while chemical sterilization markedly decreased it. Cyclic washing with sterilization by γ-irradiation present an effective processing method of the harvested human bone as it removes both the bioburden and bone marrow components. The use of a clean bone material is favorable for the production of standardized scaffolds for tissue engineering applications.

Further improvement of the bone graft processing techniques and evaluation of the resulting material for its mechanical stability as well as compatibility with human MSCs both *in vitro* and *in vivo* is needed.

## Funding

This work was supported by the Ministry of Health of the Russian Federation [grant no 115030510010].

*Conflict of interest statement*. None declared.
